# Comparative Outcomes of Left Bundle Branch Pacing and Biventricular Pacing for Cardiac Resynchronization Therapy in Heart Failure with Reduced Ejection Fraction

**DOI:** 10.3390/diagnostics16091392

**Published:** 2026-05-04

**Authors:** Fedan Hacizade, Mert Dogan, Kudret Aytemir, Ugur Canpolat

**Affiliations:** Device Unit, Department of Cardiology, Hacettepe University Faculty of Medicine, 06230 Ankara, Türkiye; fedan.hacizade@yahoo.com (F.H.); drmertd@gmail.com (M.D.); aytemirk@gmail.com (K.A.)

**Keywords:** cardiac resynchronization therapy, left bundle branch area pacing, biventricular pacing, heart failure with reduced ejection fraction, conduction system pacing

## Abstract

**Background:** Left bundle branch area pacing (LBBaP) has emerged as a physiological alternative to conventional biventricular pacing (BiVP) for cardiac resynchronization therapy (CRT). We aimed to compare long-term clinical, electrical, and echocardiographic outcomes of LBBaP versus BiVP in patients with heart failure with reduced ejection fraction (HFrEF). **Methods:** In this single-center retrospective study, 271 consecutive patients undergoing CRT implantation were included (LBBaP, *n* = 68; BiVP, *n* = 203). Outcomes included electrical resynchronization parameters, echocardiographic reverse remodeling, heart failure hospitalization, and all-cause mortality during a median follow-up of 41 months. **Results:** LBBaP achieved greater electrical resynchronization, with shorter postprocedural QRS duration (144 vs. 153 ms; *p* = 0.005) and shorter left ventricular activation time compared with BiVP. LBBaP was associated with lower radiation exposure (124 vs. 244 mGy; *p* < 0.001) and lower pacing thresholds. At 6 months, LVEF was higher in the LBBaP group (37.7% vs. 33.0%; *p* = 0.005), and heart failure hospitalization occurred less frequently (22.6% vs. 36.7%; *p* = 0.042). Long-term all-cause mortality did not differ between groups (*p* = 0.289). In multivariable analysis, baseline renal dysfunction and heart failure hospitalization within 6 months independently predicted mortality. **Conclusions:** In patients with HFrEF undergoing CRT, LBBaP provides superior electrical resynchronization and greater reverse remodeling compared with BiVP. Although associated with improved short-term clinical outcomes, long-term survival appears primarily determined by comorbid conditions rather than pacing modality.

## 1. Introduction

Heart failure with reduced ejection fraction (HFrEF) is a progressive clinical syndrome associated with substantial morbidity and mortality [1]. Cardiac resynchronization therapy (CRT) is an established treatment that improves symptoms, promotes reverse ventricular remodeling, and reduces mortality in appropriately selected patients [2]. Conventionally, CRT is delivered through biventricular pacing (BiVP); however, left bundle branch area pacing (LBBaP) has recently emerged as a physiologically based alternative with potential technical and clinical advantages [3].

Despite high implantation success rates, BiVP is limited by challenges including phrenic nerve stimulation, coronary sinus lead instability, and a nonresponse rate approaching 40% [4,5]. His bundle pacing was the first conduction system pacing approach introduced to address these limitations; however, its widespread adoption has been hindered by high pacing thresholds and lead instability [6]. LBBaP, subsequently developed as a more feasible alternative, enables reliable recruitment of the left bundle branch through deep septal lead deployment, seeking to restore physiological ventricular activation and overcome the shortcomings of conventional BiVP [6]. Although comparative evidence has rapidly expanded—including randomized and observational studies and recent meta-analyses—data on very long-term clinical outcomes and real-world durability of LBBaP/LBBP compared with conventional BiVP remain comparatively less well defined [7]. Therefore, the present study aimed to compare the long-term clinical, electrical, and echocardiographic outcomes of LBBaP versus BiVP in a real-world cohort of patients with HFrEF with heterogeneous QRS morphology and high ischemic cardiomyopathy prevalence, with specific attention to determinants of long-term survival.

## 2. Methods

### 2.1. Study Population

This single-center, retrospective observational study included patients who underwent cardiac resynchronization therapy (CRT) implantation at our institution between 1 January 2017, and 31 July 2024. A total of 455 consecutive patients undergoing CRT implantation were initially screened. Patients were stratified into two groups according to implantation strategy: left bundle branch area pacing CRT (LBBaP-CRT) and biventricular pacing CRT (BiVP-CRT). The pacing modality was selected at the operator’s discretion.

Patients were excluded if any of the following criteria were present: (1) failed transvenous CRT implantation; (2) age < 18 years; (3) follow-up duration < 6 months or unavailable follow-up data; (4) prior BiVP-CRT subsequently upgraded or converted to LBBaP-CRT; or (5) implantation of left bundle branch optimized CRT (LOT-CRT), defined as combined LBBaP and coronary sinus pacing. Based on these criteria, 184 patients were excluded, yielding a final study population of 271 patients.

Baseline demographic and clinical characteristics, including age, sex, comorbidities, electrocardiographic (ECG) and echocardiographic parameters, laboratory data, and preprocedural medical therapy, were retrieved from the institutional electronic medical records. Procedural characteristics, intracardiac electrograms (EGMs), peri-procedural complications, and device interrogation parameters were recorded. Long-term follow-up data, including device performance, ECG and echocardiographic changes, hospitalizations, and device-related complications, were obtained from hospital records and verified using the national health registry system.

The study protocol was approved by the Hacettepe University Health Sciences Ethics Committee (5 November 2024; Decision No. 2024/19-09). The requirement for informed consent was waived due to the retrospective design. The study complied with the Declaration of Helsinki and its subsequent amendments.

### 2.2. Pre-Procedural Assessment

Standard 12-lead surface ECGs were obtained before implantation to evaluate intrinsic rhythm, QRS morphology and duration, left ventricular activation time (LVAT), and atrioventricular conduction status. ECG measurements were performed digitally using electronic calipers (EP Calipers, EP Studios, Inc., Parker, CO, USA).

Transthoracic echocardiography was performed to assess left ventricular ejection fraction (LVEF), interventricular septal anatomy and thickness, left ventricular end-diastolic diameter (LVEDD), valvular disease, and cardiac chamber dimensions. LVEF was calculated using the modified Simpson method. Mitral valve area in stenotic lesions was calculated using the pressure half-time method, and aortic stenosis severity was assessed using the Bernoulli equation. Valvular regurgitation severity was visually graded as mild, moderate, or severe using color Doppler imaging [8].

Baseline laboratory evaluation included complete blood count, renal and liver function tests, B-type natriuretic peptide (BNP), and thyroid function tests.

For patients receiving warfarin, procedures were performed without interruption when the international normalized ratio (INR) was <2.5. Bridging with low-molecular-weight heparin was used when INR was <2.0, with the final dose withheld on the day of the procedure. Direct oral anticoagulants were discontinued 24–48 h prior to implantation according to renal function. All patients received prophylactic intravenous cefazolin immediately before the procedure.

### 2.3. Procedural Technique

All procedures were performed under conscious sedation using intravenous midazolam and fentanyl. Local anesthesia was achieved with prilocaine infiltration in the left pectoral region. Venous access was obtained prior to device pocket creation. Procedures were performed under fluoroscopic guidance with continuous 12-lead ECG monitoring (Artis Zee Angiography System, Siemens Healthcare GmbH, Erlangen, Germany).

### 2.4. BiVP Implantation

**Figure 1:**
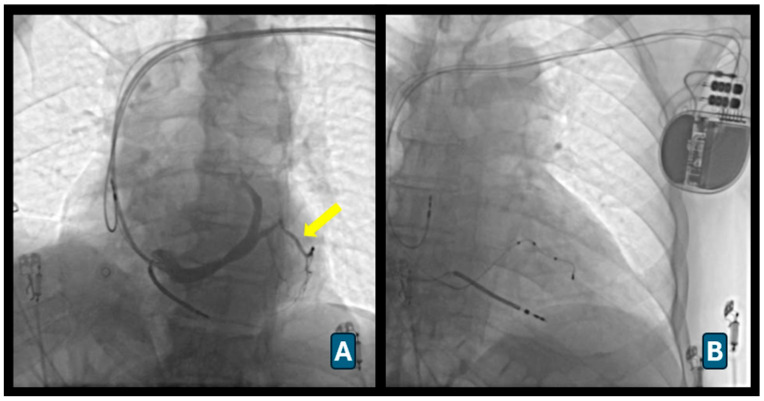
Biventricular pacing (BiVP) implantation. (**A**) Coronary sinus venography demonstrating the coronary venous anatomy and identification of the target lateral branch (arrow) for left ventricular lead placement. (**B**) Final fluoroscopic image showing successful positioning of the right atrial, right ventricular, and coronary sinus left ventricular leads connected to the cardiac resynchronization therapy device following BiVP implantation.

Right atrial leads were positioned in the atrial appendage in patients with sinus rhythm, and right ventricular leads were positioned at the apex using standard techniques. The coronary sinus was cannulated using a pre-shaped diagnostic catheter within a long peel-away sheath, followed by coronary venography to define venous anatomy (Figure 1A).

A bipolar or quadripolar left ventricular lead was advanced over a 0.014-inch guidewire into a target branch, preferably the anterolateral or posterolateral vein. Adequate sensing and pacing thresholds were confirmed, and phrenic nerve stimulation was excluded prior to sheath removal (Figure 1B). Leads were secured to the pectoral muscle and connected to the pulse generator, and the pocket was closed in layers.

### 2.5. LBBaP Implantation

**Figure 2:**
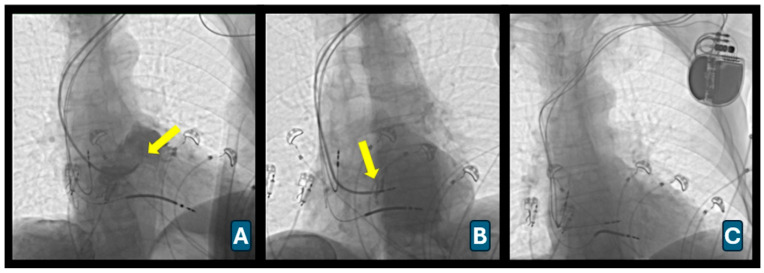
Left bundle branch area pacing (LBBaP) implantation. (**A**) Fluoroscopic right anterior oblique projection demonstrating positioning of the delivery sheath against the interventricular septum and initial lead placement site (arrow). (**B**) Advancement of the pacing lead into the interventricular septum with progressive penetration toward the left bundle branch area (arrow). (**C**) Final fluoroscopic image confirming successful LBBaP lead deployment and connection to the CRT device with stable septal positioning.

Right atrial and right ventricular leads were positioned using standard techniques. For LBBaP, a Selectra 3D delivery sheath (Biotronik, Germany) was advanced into the right ventricle over a 0.038-inch guidewire. The Solia S60 ventricular lead was prepared ex vivo by helix extension and inner coil pre-tensioning as previously described [9]. Lead deployment was guided by fluoroscopy, continuous ECG monitoring, and intracardiac electrograms recorded via a pacing system analyzer (Renamic Neo, Biotronik, Germany) [10]. The sheath was positioned against the interventricular septum in the right anterior oblique 30° view (Figure 2A). Contrast ventriculography was performed to delineate septal anatomy.

Optimal lead position was identified using unipolar pacing mapping targeting a “W” morphology in lead V1, aVL/aVR discordance, and greater R-wave amplitude in lead II than lead III. Lead depth was confirmed in the left anterior oblique projection (Figure 2B).

During the septal penetration, impedance, fluoroscopic depth, and QRS morphology were continuously monitored. After development of a terminal r/R pattern in lead V1, the following parameters were assessed:

1. V6 R-wave peak time (V6RWPT/LVAT)
2. V6–V1 interpeak interval
3. QRS transition during threshold testing
4. Presence of left bundle or fascicular potentials
5. Unipolar and bipolar pacing thresholds

Successful capture was defined according to EHRA consensus criteria for selective or nonselective left bundle branch pacing [11]. After confirmation, the sheath was removed and the lead connected to the LV port of the generator (Figure 2C). Postprocedural echocardiography was performed to confirm septal lead depth and exclude septal complications.

### 2.6. Post-Procedural Management and Follow-Up

All patients underwent chest radiography, echocardiography, 12-lead ECG, and laboratory testing following implantation and were observed for at least 24 h.

Follow-up visits were scheduled at 1 month, 6 months, and every 6 months thereafter. Each visit included clinical evaluation (NYHA functional class and congestion assessment), ECG, echocardiography, laboratory testing, and device interrogation to assess pacing thresholds, sensing amplitudes, and lead impedance.

CRT-related complications, hospitalizations, and all-cause mortality were recorded throughout follow-up.

### 2.7. Statistical Analysis

Statistical analyses were performed using SPSS Statistics version 28.0 (IBM Corp., Armonk, NY, USA). Continuous variables are presented as mean ± standard deviation or median (range) according to distribution. Categorical variables are expressed as frequencies and percentages.

Normality was assessed using visual inspection and Shapiro–Wilk or Kolmogorov–Smirnov tests. Between-group comparisons were performed using Student’s *t* test or Mann–Whitney U test for continuous variables and χ^2^ or McNemar tests for categorical variables, as appropriate. Paired comparisons were analyzed using paired *t* tests or Wilcoxon signed-rank tests.

Overall survival was defined as the interval from CRT implantation to death from any cause. Survival curves were constructed using the Kaplan–Meier method and compared using the log-rank test. Predictors of survival were assessed using univariable and multivariable Cox proportional hazards regression analyses. Variables significant in univariable analysis were entered into multivariable models after assessment for multicollinearity. A 2-sided *p* value < 0.05 was considered statistically significant.

## 3. Results

### 3.1. Patient Demographics

The study population consisted of 271 patients (mean age 63.6 ± 13.0 years; 69.4% male). CRT was delivered using biventricular pacing (BiVP) in 203 patients (74.9%) and left bundle branch area pacing (LBBaP) in 68 patients (25.1%).

Baseline demographic characteristics were comparable between groups, including age, sex, body mass index, NYHA functional class, prior cardiac implantable electronic device (CIED) implantation, and overall comorbidity burden. Ischemic cardiomyopathy was more prevalent in the BiVP group than in the LBBaP group (52.2% vs. 33.8%, *p* = 0.009). Serum creatinine levels were lower in the LBBaP group (*p* = 0.032); however, estimated glomerular filtration rate (eGFR) categories did not differ significantly between groups (*p* = 0.123).

Guideline-directed medical therapy was widely used, including beta-blockers (86.0%), loop diuretics (68.6%), mineralocorticoid receptor antagonists (56.6%), angiotensin-converting enzyme inhibitors (44.3%), and angiotensin receptor blockers (27.7%), with no between-group differences. Sodium–glucose cotransporter-2 (SGLT2) inhibitor use was significantly higher in the LBBaP group (30.9% vs. 8.9%, *p* < 0.001). Baseline characteristics are summarized in Table 1.

### 3.2. Baseline Electrocardiographic and Echocardiographic Characteristics

Persistent atrial fibrillation was present in 25.1% of patients, without differences between groups (*p* = 0.531). Baseline QRS duration was similar between BiVP and LBBaP groups (171 ± 29 ms vs. 170 ± 30 ms; *p* = 0.706). Left bundle branch block was the predominant morphology (44.5%), with a trend toward lower prevalence in the LBBaP group (34.3% vs. 48.1%; *p* = 0.051). Left anterior hemiblock (*p* = 0.015) and first-degree atrioventricular block (*p* = 0.038) were more frequent in the LBBaP group. Left ventricular activation time (LVAT) did not differ significantly; however, the LVAT/QRS ratio was lower in the LBBaP group (0.59 ± 0.07 vs. 0.61 ± 0.07; *p* = 0.011).

Baseline echocardiographic parameters were comparable between groups, including left ventricular ejection fraction (LVEF) (29 ± 7.2%) and left ventricular end-diastolic diameter (LVEDD) (6.0 ± 0.8 cm) (*p* = 0.093 and *p* = 0.115, respectively). Moderate-to-severe mitral regurgitation was more common in the BiVP group (70.3% vs. 55.9%; *p* = 0.029), whereas tricuspid and aortic regurgitation rates were similar.

Detailed electrocardiographic and echocardiographic data are presented in Table 2.

### 3.3. Procedural Outcomes and Complications

Median procedure duration (84 min) and fluoroscopy time (13.9 min) were similar between groups (*p* = 0.698 and *p* = 0.563, respectively). Radiation exposure was significantly lower in the LBBaP group (124 mGy vs. 244 mGy; *p* < 0.001). LBBaP demonstrated superior electrical performance, including lower pacing thresholds (*p* < 0.001), lower lead impedance (*p* < 0.001), and absence of pulse width requirements > 0.4 ms (0% vs. 9.9%; *p* = 0.007). Crossover occurred in 4.8% of patients (*p* = 0.528). Eleven patients in the LBBaP group crossed over to BiVP due to septal perforation, inability to penetrate the septum, or suboptimal electrical parameters. Two BiVP cases crossed over to LBBaP because of unfavorable coronary venous anatomy.

Postprocedural QRS duration was shorter in the LBBaP group (144 ± 18 ms vs. 153 ± 22 ms; *p* = 0.005), with greater QRS reduction (27 ms vs. 17 ms; *p* = 0.017). LVAT was also shorter in the LBBaP group (81.3 ± 13.3 ms vs. 93.4 ± 16.0 ms; *p* < 0.001).

The overall complication rate was 13.3%, without significant between-group differences. Acute complications requiring intervention within 24 h occurred in 1.5% of patients, including pneumothorax, lead dislodgement, and asystole. An additional 2.6% were managed conservatively. During the first 6 months, device-related complications occurred in 9.2% of patients, most commonly pocket hematoma (4.1%) and lead dislodgement (4.4%), with no differences between groups.

Detailed outcomes are presented in Table 3.

### 3.4. Six-Month and Long-Term Clinical Outcomes

At 6 months, functional status improved more prominently in the LBBaP group, with a higher proportion of patients in NYHA class I (32.1% vs. 12.5%) and fewer in class III (14.3% vs. 27.1%) (*p* = 0.006). Electrical resynchronization remained superior with LBBaP. Paced QRS duration at 6 months was shorter (146 ± 19 ms vs. 153 ± 19.3 ms; *p* = 0.017), and QRS reduction was greater (31 ms vs. 19 ms; *p* = 0.036). LVAT and LVAT/QRS ratio were also lower (both *p* < 0.001). Echocardiographically, LVEF was higher in the LBBaP group (37.7 ± 10.8% vs. 33.0 ± 10.6%; *p* = 0.005). A greater proportion achieved LVEF ≥ 40% (50.9% vs. 28.4%; *p* = 0.003). LBBaP was also associated with reduced LVEDD (*p* = 0.025) and lower prevalence of moderate-to-severe mitral regurgitation (32.1% vs. 54.3%; *p* = 0.005).

Pacing thresholds and impedance remained lower in the LBBaP group at 6 months (*p* < 0.001). Heart failure hospitalization within 6 months occurred less frequently in the LBBaP group (22.6% vs. 36.7%; *p* = 0.042). Six-month all-cause mortality was also lower (2.9% vs. 10.8%; *p* = 0.047).

Subgroup analyses according to cardiomyopathy etiology revealed distinct patterns of response. In the ischemic cardiomyopathy subgroup (BiVP *n* = 106, LBBaP *n* = 23), LBBaP was associated with significantly shorter LVAT (82.8 ± 13.7 ms vs. 92.7 ± 13.8 ms; *p* = 0.004), higher LVEF (36 ± 8% vs. 30 ± 10%; *p* = 0.014), smaller LVEDD (5.5 ± 0.7 cm vs. 6.0 ± 0.9 cm; *p* = 0.009), lower pacing thresholds (0.55 vs 0.75 V; *p* = 0.021), lower impedance (346 vs 527 ohm; *p* < 0.001), and lower heart failure hospitalization rates (13.6% vs 41.3%; *p* = 0.015). In the non-ischemic cardiomyopathy subgroup (BiVP *n* = 97, LBBaP *n* = 45), LBBaP demonstrated superior electrical resynchronization with shorter paced QRS duration (144 ± 20 ms vs 154 ± 21 ms; *p* = 0.029), shorter LVAT (82.6 ± 13.7 ms vs 91.2 ± 13.7 ms; *p* = 0.003), lower pacing thresholds (0.55 vs 0.75 V; *p* < 0.001), and lower impedance (385 vs 570 ohm; *p* < 0.001); however, echocardiographic and clinical outcomes did not differ significantly between groups. Detailed outcomes are presented in Table 4.

### 3.5. Long-Term Survival Analysis

**Figure 3:**
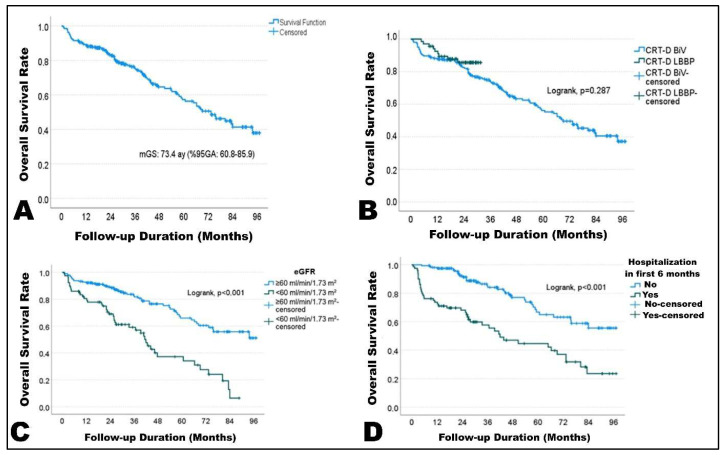
Long-term survival analyses. (**A**) Kaplan–Meier curve showing overall survival of the entire study cohort during follow-up (median overall survival 73.4 months; 95% CI 60.8–85.9). (**B**) Kaplan–Meier survival comparison between patients receiving BiVP and LBBaP CRT, demonstrating no significant difference in long-term survival (log-rank *p* = 0.287). (**C**) Kaplan–Meier survival stratified by baseline renal function, showing significantly reduced survival in patients with estimated glomerular filtration rate (eGFR) < 60 mL/min/1.73 m^2^ (log-rank *p* < 0.001). (**D**) Kaplan–Meier survival according to heart failure hospitalization within the first 6 months after CRT implantation, demonstrating significantly worse survival among hospitalized patients (log-rank *p* < 0.001).

During a median follow-up of 41 months (95% CI: 33.9–48.2), 95 patients (35.1%) died. Median overall survival was 73.4 months (95% CI: 60.8–85.9). Estimated survival rates were 82.8% at 2 years and 57.3% at 5 years (Figure 3A).

In univariable Cox regression analysis, predictors of mortality included age, ischemic cardiomyopathy, higher NYHA class, renal dysfunction, and hospitalization within the first 6 months. Pacing modality (LBBaP vs. BiVP) was not independently associated with long-term mortality (HR 0.678; *p* = 0.289) (Figure 3B). Multivariable Cox regression identified baseline eGFR < 60 mL/min/1.73 m^2^ (HR 2.495; *p* < 0.001) and hospitalization within the first 6 months (HR 1.915; *p* = 0.010) as independent predictors of mortality (Figure 3C,D). Other variables, including age and baseline LVEF, were not independently associated with mortality after adjustment.

## 4. Discussion

### 4.1. Main Findings

In this single-center retrospective study, we compared long-term clinical outcomes of left bundle branch area pacing (LBBaP)–CRT and biventricular pacing (BiVP)–CRT in patients with heart failure with reduced ejection fraction. The principal findings were the following: (1) LBBaP achieved superior electrical resynchronization, reflected by shorter QRS duration and left ventricular activation time (LVAT); (2) at 6 months, LBBaP was associated with greater improvement in left ventricular ejection fraction, fewer heart failure hospitalizations, and lower early mortality; (3) during long-term follow-up, pacing modality was not independently associated with all-cause mortality; (4) baseline renal dysfunction and early heart failure hospitalization were the strongest predictors of long-term survival.

The present study offers several distinctive contributions relative to existing evidence. Unlike recent randomized trials that enrolled exclusively patients with LBBB morphology and predominantly non-ischemic cardiomyopathy [12,13], our cohort reflects a broader real-world population: LBBB was present in only 44.5% of patients, and ischemic cardiomyopathy accounted for nearly half of the cases. Furthermore, the extended follow-up of 41 months enables the characterization of long-term survival determinants that are not captured in shorter-term analyses.

### 4.2. Physiological Pacing and Electrical Resynchronization

Limitations inherent to conventional BiVP have accelerated interest in physiological pacing strategies. By directly recruiting the His–Purkinje system, LBBaP enables near-native ventricular activation and avoids anatomical constraints associated with coronary sinus lead placement. Consistent with accumulating evidence, our findings demonstrate superior electrical resynchronization with LBBaP, characterized by narrower QRS complexes and shorter LVAT, together with stable and lower pacing thresholds [14].

Importantly, this study extends prior observations by demonstrating that these electrical advantages translate into sustained clinical and structural benefits during extended follow-up. Although further multicenter randomized studies are required, our results support conduction system pacing as a mechanistically sound alternative for CRT delivery.

### 4.3. Electrical Substrate and Patient Complexity

Our cohort exhibited longer baseline QRS duration (171 ± 21 ms) compared with pivotal studies such as I-CLAS (156 ± 29 ms) [15] and reports by Shroff et al. [16] and Vijayaraman et al. [17] (~160 ms), suggesting a population with more advanced electrical dyssynchrony. Additionally, the prevalence of left bundle branch block in the LBBaP group was relatively low (34.3%) compared with previously reported cohorts [15,17,18]

Despite a higher proportion of non-specific intraventricular conduction delay patterns, LBBaP achieved greater QRS narrowing and shorter LVAT than BiVP. These findings align with our previous work demonstrating preservation of ventricular depolarization and repolarization dynamics following LBBaP, supporting the concept that recruitment of the native conduction system enables more homogeneous ventricular activation independent of baseline conduction morphology.

### 4.4. Procedural Performance and Safety

Procedural duration was comparable between groups, consistent with prior studies [17,19]. Notably, radiation exposure was significantly lower with LBBaP. This difference likely reflects the technical complexity of coronary sinus cannulation and branch selection during BiVP implantation, which frequently requires prolonged fluoroscopic guidance.

Complication rates were similar between groups, corroborating existing safety data [15,20]. Physiological pacing may also contribute to electrical stability, as previously suggested by our observations regarding QTc behavior following LBBaP implantation [21], potentially explaining the low incidence of arrhythmic complications.

### 4.5. Reverse Remodeling and Clinical Outcomes

The superior electrical resynchronization achieved with LBBaP translated into greater reverse remodeling at 6 months, with a higher proportion of patients achieving LVEF ≥ 40%, and significant reductions in LVEDD and mitral regurgitation severity. Although the magnitude of LVEF improvement was modest compared with some reports, [22,23] this likely reflects the complexity of our cohort, characterized by wider baseline QRS duration and lower LBBB prevalence.

The higher use of SGLT2 inhibitors in the LBBaP group represents a potential confounder that may have contributed to improved outcomes. Nevertheless, the consistent association between electrical resynchronization parameters and structural improvement suggests that restoration of physiological activation remained the primary driver of remodeling.

Randomized data support the efficacy of LBBP for CRT. In the LBBP-RESYNC trial, LBBP-CRT achieved greater improvement in LV function compared with BiVP-CRT in patients with nonischemic cardiomyopathy and LBBB at 6 months [7]. In addition, a meta-analysis by Parlavecchio et al., including 10 studies and >1000 patients reported that LBBP-CRT was associated with improved clinical and electrical/echocardiographic outcomes, including fewer heart-failure hospitalizations compared with BiVP-CRT [24]. These findings are consistent with our observed reductions in early heart-failure hospitalization and improved reverse remodeling, while our extended follow-up provides additional perspective on longer-term survival signals

### 4.6. Determinants of Long-Term Survival

Despite favorable short-term outcomes, pacing modality was not independently associated with long-term mortality. Instead, renal dysfunction and early heart failure hospitalization emerged as dominant predictors of survival. This observation suggests that correction of electrical dyssynchrony alone may not fully modify long-term prognosis, which appears strongly influenced by systemic disease burden.

The prognostic impact of hospitalization within the first 6 months highlights this interval as a vulnerable phase following CRT implantation, consistent with previous CRT cohorts [17,20]. These findings emphasize the importance of intensified clinical surveillance, early optimization of guideline-directed medical therapy, and proactive device management during early follow-up.

On the other hand, these findings appear to contrast with a recent meta-analysis reporting a significant reduction in all-cause mortality with LBBAP (HR 0.83; 95% CI 0.71–0.96) [25]. However, several considerations contextualize this discrepancy. The median follow-up in that pooled analysis was only 13.8 months—substantially shorter than the 41 months in our cohort—and the mortality benefit was statistically significant only in retrospective subgroups, not in prospective analyses. Furthermore, the pooled estimate was sensitive to the exclusion of a single study. Importantly, the HeartSync-LBBP randomized trial—the largest prospective randomized trial to date—similarly found no significant difference in all-cause mortality between LBBP and BiVP (HR 0.40; *p* = 0.25) [12], consistent with our findings. Collectively, these observations suggest that while LBBaP improves heart failure hospitalization rates and promotes superior reverse remodeling, its capacity to independently modify long-term survival may be attenuated in populations with significant competing comorbidity risk.

### 4.7. Impact of Ischemic Etiology

The higher prevalence of ischemic cardiomyopathy in the BiVP group represents an important baseline imbalance. Myocardial scar burden is a well-recognized determinant of CRT response and may partly explain the lower degree of reverse remodeling and higher hospitalization rates observed in this group [26]. Nevertheless, comparable long-term survival between groups suggests that systemic comorbidities, particularly renal dysfunction, exert a greater influence on prognosis than pacing modality alone.

Subgroup analyses revealed an unexpected pattern: in the ischemic cardiomyopathy subgroup, LBBaP was associated with superior outcomes across both electrical and clinical domains, including higher LVEF, smaller LVEDD, and lower heart failure hospitalization rates, whereas in the non-ischemic subgroup, LBBaP demonstrated consistent electrical superiority without significant echocardiographic or clinical differences at 6 months. These findings diverge from the conventional expectation that non-ischemic cardiomyopathy would be more amenable to resynchronization therapy [26]. Several factors may explain this pattern. The non-randomized design allowed preferential selection of anatomically favorable patients for LBBaP in the ischemic subgroup, with crossovers to BiVP in cases of suboptimal electrical response, potentially enriching this group with more favorable responders. Additionally, the small ischemic LBBaP subgroup (n = 23) limits the reliability of these comparisons. Furthermore, emerging evidence suggests that myocardial scar burden critically modulates CRT response independent of pacing modality—high scar burden attenuates LBBaP benefit even in non-ischemic patients [27]. Since cardiac MRI was not routinely performed, septal scar could not be assessed, and occult fibrosis in the non-ischemic subgroup may have blunted the clinical benefit of LBBaP. These findings underscore the importance of myocardial substrate characterization prior to CRT implantation.

## 5. Limitations

This study has several limitations. First, its retrospective single-center design introduces potential selection bias and residual confounding related to operator-dependent device selection. Second, although adequately powered for primary analyses, subgroup analyses—particularly according to QRS morphology—may have been underpowered. Third, echocardiographic follow-up was standardized at 6 months and therefore may not fully capture long-term remodeling trajectories. Fourth, detailed electrophysiological characterization distinguishing selective from nonselective LBB capture and post-implant programming optimization were not systematically available. Fifth, the significantly higher use of SGLT2 inhibitors in the LBBaP group likely reflects a temporal confounding effect, as LBBaP was introduced at our center in the latter part of the study period, coinciding with the broader adoption of SGLT2 inhibitors into guideline-directed medical therapy for HFrEF. This imbalance may have contributed to the more favorable short-term outcomes observed in the LBBaP group and should be considered when interpreting the results. Finally, objective functional capacity assessments such as cardiopulmonary exercise testing or 6-minute walk testing were not routinely performed.

## 6. Conclusions

In patients with heart failure with reduced ejection fraction and wide QRS complexes, LBBaP provides superior electrical resynchronization, greater reverse remodeling, and lower radiation exposure compared with conventional BiVP. Although associated with improved short-term clinical outcomes, long-term survival was primarily determined by renal function and early clinical stability rather than pacing modality. LBBaP represents a safe and effective physiological alternative for delivery of cardiac resynchronization therapy.

## Figures and Tables

**Table 1:** Baseline demographic, clinical, and laboratory characteristics of the study population.

Characteristics	Total (*n* = 271)	BiVP (*n* = 203)	LBBaP (*n* = 68)	*p*-Value
Demographics				
Age, years (mean ± SD)	63.6 ± 13.0	64.1 ± 13.1	62.4 ± 12.6	0.353
Male Sex, *n* (%)	188 (69.4)	147 (72.4)	41 (60.3)	0.061
BMI, kg/m^2^ (mean ± SD)	26.9 ± 4.7	26.9 ± 4.6	26.7 ± 4.8	0.692
Clinical Characteristics				
Etiology, *n* (%)				0.009
Ischemic CMP	129 (47.6)	106 (52.2)	23 (33.8)	
Non-ischemic CMP	142 (52.4)	97 (47.8)	45 (66.2)	
NYHA Functional Class, *n* (%)				0.149
Class I-II	133 (49.1)	95 (46.8)	38 (55.9)	
Class III-IV	138 (50.9)	108 (53.2)	30 (44.1)	
Prior CIED History, *n* (%)	90 (33.2)	70 (34.5)	20 (29.4)	0.442
Comorbidities, *n* (%)				
Hypertension	201 (74.2)	152 (74.9)	49 (72.1)	0.646
Diabetes Mellitus	158 (58.3)	120 (59.1)	38 (55.9)	0.640
Cerebrovascular Disease	35 (12.9)	29 (14.3)	6 (8.8)	0.245
Renal Failure (Dialysis)	6 (2.2)	4 (2.0)	2 (2.9)	0.643
Medical Therapy, *n* (%)				
Beta-blockers	233 (86.0)	175 (86.2)	58 (85.3)	0.851
ACEI/ARB/ARNI	219 (80.8)	166 (81.8)	53 (77.9)	0.482
MRA	154 (56.6)	109 (53.7)	45 (66.2)	0.072
SGLT-2 Inhibitors	39 (14.4)	18 (8.9)	21 (30.9)	<0.001
Loop Diuretics	186 (68.6)	139 (68.5)	47 (69.1)	0.921
DOAC/Warfarin	90 (33.2)	67 (33.0)	23 (33.8)	0.901
Amiodarone	31 (11.4)	25 (12.3)	6 (8.8)	0.434
Digoxin	42 (15.5)	33 (16.3)	9 (13.2)	0.551
Laboratory Findings				
Hemoglobin, g/dL	13.0 ± 1.9	13.0 ± 1.9	12.9 ± 1.8	0.491
Serum creatinine, mg/dL *	0.96 (0.4–6)	0.97 (0.4–6)	0.91 (0.5–3.5)	0.032
BNP, pg/mL *	409 (10–7027)	448 (10–7027)	347 (10–6018)	0.282

Angiotensin-converting enzyme inhibitor; ARB: Angiotensin receptor blocker; ARNI: Angiotensin receptor-neprilysin inhibitor; BiVP: Biventricular pacing; BMI: Body mass index; BNP: B-type natriuretic peptide; CIED: Cardiac implantable electronic device; CMP: Cardiomyopathy; DOAC: Direct oral anticoagulant; LBBaP: Left bundle branch area pacing; MRA: Mineralocorticoid receptor antagonist; NYHA: New York Heart Association; SD: Standard deviation; SGLT-2: Sodium–glucose cotransporter-2. * Values are presented as median (minimum-maximum).

**Table 2:** Baseline electrocardiographic and echocardiographic characteristics of the study population.

Parameters	Total (*n* = 271)	BiVP (*n* = 203)	LBBaP (*n* = 68)	*p*-Value
Electrocardiographic Findings				
Rhythm, *n* (%)				
Persistent AF	68 (25.1)	49 (24.1)	19 (27.9)	0.531
Paroxysmal AF	10 (3.7)	7 (3.4)	3 (4.4)	0.715
Paced Rhythm	49 (18.1)	34 (16.7)	15 (22.1)	0.325
QRS Duration, ms (mean ± SD)	171 ± 29	171 ± 29	170 ± 30	0.706
QRS Morphology, *n* (%)				
LBBB	114 (44.5)	91 (48.1)	23 (34.3)	0.051
LAHB	7 (2.7)	2 (1.1)	5 (7.5)	0.015
RBBB	11 (4.3)	7 (3.7)	4 (6.0)	0.485
IVCD	71 (27.7)	53 (28.0)	18 (26.9)	0.853
AV Conduction, *n* (%)				
1st Degree AV Block	20 (7.6)	11 (5.6)	9 (13.4)	0.038
2nd Degree AV Block	7 (2.7)	5 (2.6)	2 (3.0)	1.000
Complete AV Block	53 (20.2)	36 (18.5)	17 (25.4)	0.224
Activation Intervals				
LVAT, ms (mean ± SD)	104 ± 21.5	105 ± 21	100 ± 22	0.097
LVAT/LVEDD ratio	17.4 ± 4.3	17.5 ± 4.1	17.3 ± 4.6	0.722
LVAT/QRS Duration ratio	0.61 ± 0.07	0.61 ± 0.07	0.59 ± 0.07	0.011
Echocardiographic Findings				
LVEF, % (mean ± SD)	29 ± 7.2	29 ± 7	30 ± 7.5	0.093
LVEDD, cm (mean ± SD)	6.0 ± 0.8	6.1 ± 0.7	5.9 ± 0.9	0.115
LA Diameter, cm (mean ± SD)	4.5 ± 0.8	4.6 ± 0.8	4.4 ± 0.8	0.277
Valvular Pathology (Mod-Severe), *n* (%)				
Mitral Regurgitation	180 (66.7)	142 (70.3)	38 (55.9)	0.029
Tricuspid Regurgitation	142 (52.6)	107 (53.0)	35 (51.5)	0.830
Aortic Regurgitation	15 (5.6)	11 (5.4)	4 (5.9)	1.000

Values are presented as *n* (%) or mean ± standard deviation. AF: Atrial Fibrillation; LBBB: Left Bundle Branch Block; LAHB: Left Anterior Hemiblock; RBBB: Right Bundle Branch Block; IVCD: Intraventricular Conduction Delay; AV: Atrioventricular; LVAT: Left Ventricular Activation Time; LVEF: Left Ventricular Ejection Fraction; LVEDD: Left Ventricular End-Diastolic Diameter; LA: Left Atrium.

**Table 3:** Procedural outcomes, electrical parameters, and complications of the study population.

Parameters	Total (*n* = 271)	BiVP (*n* = 203)	LBBaP (*n* = 68)	*p*-Value
Procedural Metrics				
Procedure Time, min [Median (min-max)]	84 (40–208)	83 (40–208)	85 (40–166)	0.698
Fluoroscopy Time, min [Median (min-max)]	13.9 (1.2–68.4)	13.7 (4.8–68.4)	14.8 (1.2–49.9)	0.563
Radiation Dose, Gy [Median (min-max)]	188 (23–1217)	244 (38–1217)	124 (23–1063)	<0.001
Crossover Rate, *n* (%)	13 (4.8)	11 (5.4)	2 (2.9)	0.528
Electrical Parameters				
Pacing Threshold, V [Median (min-max)]	0.85 (0.3–6)	0.9 (0.3–6)	0.7 (0.3–2.2)	<0.001
Impedance, Ohm [Median (min-max)]	596 (304–1790)	640 (304–1790)	507 (306–980)	<0.001
Pulse Width > 0.4 ms, *n* (%)	20 (7.4)	20 (9.9)	0 (0.0)	0.007
ECG Outcomes				
Post-proc QRS Duration, ms (mean ± SD)	151 ± 21	153 ± 22	144 ± 18	0.005
ΔQRS (Reduction), ms [Median (min-max)]	20 (−47–102)	17 (−47–102)	27 (−24–72)	0.017
Post-proc LVAT, ms (mean ± SD)	90.3 ± 16.2	93.4 ± 16.0	81.3 ± 13.3	<0.001
Laboratory Safety				
Post-proc Hemoglobin, g/dL (mean ± SD)	12.7 ± 1.9	12.8 ± 2.0	12.2 ± 1.8	0.031
Post-proc Creatinine, mg/dL [Median (min-max)]	0.96 (0.4–7.3)	0.99 (0.4–7.3)	0.91 (0.4–3.6)	0.013
Complications, *n* (%)				
Total Complications	36 (13.3)	28 (13.8)	8 (11.8)	0.670
Acute (<24h) Requiring Intervention	4 (1.5)	3 (1.5)	1 (1.5)	1.000
Subacute (24h-6mo) Device-Related	25 (9.2)	19 (9.4)	6 (8.8)	0.895
Pocket Hematoma	11 (4.1)	7 (3.4)	4 (5.9)	0.476
Lead Dislodgment	12 (4.4)	10 (4.9)	2 (2.9)	0.736

Values are presented as *n* (%), mean ± standard deviation, or median (minimum-maximum). BiVP: Biventricular Pacing; LBBaP: Left Bundle Branch Area Pacing; LVAT: Left Ventricular Activation Time; Gy: Gray.

**Table 4:** Clinical, echocardiographic, and electrical outcomes at 6-month follow-up.

Parameters	Total (*n* = 271)	BiVP (*n* = 203)	LBBaP (*n* = 68)	*p*-Value
NYHA Functional Class, *n* (%)				0.006
Class I	36 (18.0)	18 (12.5)	18 (32.1)	
Class II	115 (57.5)	85 (59.0)	30 (53.6)	
Class III	47 (23.5)	39 (27.1)	8 (14.3)	
Class IV	2 (1.0)	2 (1.4)	0 (0.0)	
Echocardiographic Outcomes				
LVEF, % (Mean ± SD)	34.4 ± 10.8	33.0 ± 10.6	37.7 ± 10.8	0.005
LVEF ≥ 40%, *n* (%)	69 (34.8)	40 (28.4)	29 (50.9)	0.003
LVEDD, cm (Mean ± SD)	5.8 ± 0.9	5.9 ± 0.8	5.6 ± 1.0	0.025
Moderate-Severe MR, *n* (%)	94 (48.0)	76 (54.3)	18 (32.1)	0.005
Electrical Outcomes				
QRS Duration, ms (Mean ± SD)	151 ± 19.5	153 ± 19.3	146 ± 19	0.017
ΔQRS, ms (Mean ± SD)	22	19	31	0.036
LVAT, ms (Mean ± SD)	89.2 ± 14.3	92.0 ± 13.7	82.6 ± 13.6	<0.001
Pacing Threshold, V (Median)	0.8	0.8	0.6	<0.001
Impedance, Ohm (Median)	519	570	375	<0.001
Clinical Events				
Hospitalization (<6 mo), *n* (%)	80 (33.1)	66 (36.7)	14 (22.6)	0.042
All-cause Mortality (<6 mo), *n* (%)	24 (8.9)	22 (10.8)	2 (2.9)	0.047

Values are presented as *n* (%), mean ± standard deviation, or median. BiVP: Biventricular Pacing; LBBaP: Left Bundle Branch Area Pacing; LVEF: Left Ventricular Ejection Fraction; MR: Mitral Regurgitation.

## Data Availability

Data are available on request from the authors.

## Abbreviations

AF	Atrial Fibrillation
AV	Atrioventricular
BiVP	Biventricular Pacing
BMI	Body Mass Index
BNP	B-type Natriuretic Peptide
CIED	Cardiac Implantable Electronic Device
CRT	Cardiac Resynchronization Therapy
CSP	Conduction System Pacing
HFrEF	Heart Failure with Reduced Ejection Fraction
LAHB	Left Anterior Hemiblock
LBBaP	Left Bundle Branch Area Pacing
LBBB	Left Bundle Branch Block
LOT-CRT	Left Bundle Branch Optimized
CRT LVAT	Left Ventricular Activation Time
LVEDD	Left Ventricular End-Diastolic Diameter
LVEF	Left Ventricular Ejection Fraction
NYHA	New York Heart Association
RBBB	Right Bundle Branch Block
